# Improvement of Sensorial, Physicochemical, Microbiological, Nutritional and Fatty Acid Attributes and Shelf Life Extension of Hot Smoked Half-Dried Pacific Saury (*Cololabis saira*)

**DOI:** 10.3390/foods9081009

**Published:** 2020-07-27

**Authors:** Md. Abdul Baten, Na Eun Won, Jae Hak Sohn, Jin-Soo Kim, Md. Mohibbullah, Jae-Suk Choi

**Affiliations:** 1 Department of Fishing and Post Harvest Technology, Sher-e-Bangla Agricultural University, Dhaka 1207, Bangladesh; mabaten.fpht@sau.edu.bd; 2 Seafood Research Center, Silla University, #605, Advanced Seafood Processing Complex, Wonyang-ro, Amnam-dong, Seo-gu, Busan 49277, Korea; ftrnd2@silla.ac.kr (N.E.W.); jhsohn@silla.ac.kr (J.H.S.); 3 Department of Food Biotechnology, Division of Bioindustry, College of Medical and Life Sciences, Silla University, Busan 46958, Korea; 4 Department of Seafood and Aquaculture Science, Gyeongsang National University, Tongyeong-si 53064, Korea; jinsukim@gnu.ac.kr

**Keywords:** hot smoking of fish, sensory evaluation, physicochemical properties, microbiology and nutrition

## Abstract

Half-dried Pacific saury of *Cololabis saira* (HDPS) is a fatty fish of high nutritional value with remarkable consumer interest in the Asia Pacific region, however, it undergoes various deteriorative changes associated with browning, bacterial contamination, oxidation, and decreased sensory attributes while marketed in various processed forms. To withstand these complications, research aimed to investigate the hot smoking technology to improve physicochemical, microbiological, and sensory attributes of HDPS with prolonged shelf life in storage conditions. The HDPS fillets were processed with hot smoking (70 °C) using various sawdust materials of Apple, Chestnut, Oak, Cherry, and Walnut, wherein the smoke time was set at different time points of 0, 20, 25, and 30 min. The results indicated that 25 min of smoking time with the selective Oak sawdust showed better sensorial characteristics, physicochemical properties, and microbiological qualities. Moreover, HDPS possessed higher nutritional value and valuable functional fatty acids, particularly docosahexaenoic acid and eicosapentaenoic acid, having a storage ability of up to 30 days at 10 °C. The processed HDPS offered a reduced level of Trimethylamine-N-oxide and Benzo[a]pyrene contents, indicating the acceptable and safe for human consumption. Therefore, HDPS with hot smoking could likely be a promising technique for preserving the premium quality of the product by providing desired characteristics of health and nutrition to end-point consumers.

## 1. Introduction

Worldwide, fish are highly valuable and nutritious food items, and there is growing interest in fish consumption as they are high in protein with a plethora of omega-3 and -6 polyunsaturated fatty acids, which are essential for human to maintain normal body functions [1]. Considering such various health facts of consuming fish and fish products, fish processors have been searching for a technique that is more effective than traditional ones, by which a fish with safety, nutrition, and delicacy of taste can be precisely ensured [2]. The Pacific saury, *Cololabis saira* (Brevoort, 1856), is a member of the family Scomberesocidae and is also known as mackerel pike or skipper [3]. It is a widely distributed fish species ranging from subarctic to subtropical regions throughout the Northwestern Pacific Ocean [4]. It is a highly migratory species and is mostly caught by countries such as Japan, China, Chinese Taipei, Russia, South Korea, and Vanuatu [5]. The Pacific saury is a commercially important fish species, especially in South Korea and traditionally known as Gwamegi in the Korean food industry [6]. This fish species is marketed in raw, frozen, and processed forms, including semi-dry, seasoned dry, and marinated products.

Smoking is a traditional fish preservation method, having remarkable economic importance worldwide [7], which is associated with combined treatment as followed by the salting, drying, and heat treatment [8]. Nowadays, smoked fish and fishery products have shown greater appeal to the consumer due to their delicacy in taste, aroma, and color [9]. Smoking treatment preserves food mainly because of the synergistic effects of temperature, table salt, and other chemical substances released from sawdust with a reduced water activity [10]. Moreover, smoking treatment prevents the growth of microbes and delays the oxidative changes in food materials [11]. The incomplete burning of wood or sawdust helps to release the volatile chemical compounds deposited on fish food surfaces, and, thereby, the process limits bacterial growth [12]. Smoking technology maintains good physicochemical characteristics of foods, such as pH level, volatile basic nitrogen (VBN) level, thiobarbituric acid reactive substances (TBARS) level, textural characteristics, and fatty acid profile and eventually improves sensory quality to the end-point consumer with extended shelf life [13]. Currently, the two types of smoking methods have been practiced for processing and preservation of fishery products; cold smoking (30 °C), smoking without thermal breakdown [14], and hot smoking (70–80 °C), with a thermal breakdown [15]. Among these, the hot smoked processing products are considered to be more acceptable by the consumer over cold-smoked ones, because of delicacy in color and attractive appearance [10].

Despite the popularity of using hot smoking in the preservation of fish and fishery products, the underlying mechanism of interactions between wood-derived chemicals and the food matrix which achieve its sensorial, physicochemical, microbiological, nutritional, and fatty acid profiling attributes is rudimentary. The hot smoke method has been employed for 70–80 °C heat treatment and results in the cooking of fish with considerable palatability, thus making it suitable for direct consumption [10]. The quality of smoked fishery product was assessed using (1) sensory evaluation based on color, texture, odor, flavor, and overall preference; (2) physicochemical parameters such as textural properties and levels of pH, VBN, TBARS, Trimethylamine-N-oxide (TMAO), and fatty acid profile; (3) nutritional value, including calories, protein, lipid, and minerals; and (4) microbial-growth estimation [16,17]. Although, there are a wide variety of studies that have examined the effect the hot smoking method has on the physicochemical composition of different fish species [7,18,19,20,21]. However, there has been no study yet performed on the effect of the hot smoking treatment in half-dried Pacific saury (HDPS). Therefore, the current study aimed to provide scientific rationality when choosing a hot smoking treatment to improve the sensory, physicochemical, microbiological, nutritional, and shelf life extension attributes of half-dried Pacific Saury (*Cololabis saira*).

## 2. Materials and Methods

### 2.1. Collection and Preparation of Sample

Half dried Pacific Saury (*Cololabis saira*) was collected from raw fish markets of South Korea, stored on ice flakes immediately after collection, and washed with tap water before filleting. The fish was filleted in lengthwise and any unwanted parts, such as scales and stomach remaining in the fillet, were carefully removed. The diameter was maintained as 14.7 × 2.6 × 0.3 cm, of which, the average weight was 22.8 ± 0.77 g. Then, the prepared fish sample was divided into two groups such as raw and smoked samples.

### 2.2. Hot Smoking Treatment

Smoking treatment is performed electronically and the temperature was fixed at 70 °C for different time-dependent treatment groups followed by 0, 10, 15, 20, 25, and 30 min of smoking. The smoke generated from different smoking materials was of Apple, Oak, Chestnut, Walnut, and Cherry, and the procedures for the preparation of hot smoking HDPS were followed by our previous reports [16,17].

### 2.3. Analysis of Sensorial Characteristics

The sensory analysis was evaluated by recruiting the trained 10 panelists with ages between 25 and 40, and the sensory parameters were considered to be the odor, color, flavor, and overall preference of raw and smoked HDPS products [16,17]. During this period, all of the experimental samples were unbiasedly encoded. Then, 10 g of the sample was given to each panelist and they were requested to score using a numerical number on a hedonic scale, where 1 stands for “extremely dislike” and 9 for “extremely like” [22].

### 2.4. Analysis of Physical Properties

The method of Goulas and Kontominas [14] was followed to determine the weight loss of the fish fillets.

The odor intensity of the processed HDPS fillet was determined at a different smoking time intervals. The 5g sample was taken from different experimental groups, kept in a 50 mL conical tube, and covered with parafilm. Upon closing the lid, the odor intensity was measured instrumentally (XP-329, New Cosmos Electric Co. Ltd., Osaka, Japan) [23].

According to Mohibbullah et al. [16] and Chen et al. [24], the color of the surface of each HDPS sample was assessed numerically with an instrument of CM-700d, Konica Minolta (Tokyo, Japan).

The smoked sample from each group was taken for texture analysis using a Brookfield Texture Analyzer (Massachusetts, USA) and equipped with a software system connecting to the computer (Texture PRO CT, Middleboro, MA, USA). Data processing and collection were followed by the study of Ganesan & Benjakul [25].

### 2.5. Analysis of Biochemical Properties

A serial dilution method was used to analyze the total bacterial count, where 9 g of sample was mixed with 45 mL sterile saline solution and homogenized. After that, the diluted samples (10^−1^, 10^−2^, and 10^−3^) were spread, incubated, and counted the observed colony, following the report of Chen et al. [24]. The total coliform count of fish samples was estimated by following the methods of the US Food and Drug Administration (FDA). The positive results were considered as sadZ gas production observed in the EC culture medium.

A 2 g HDPS fillet sample from each treatment group was taken with 18 mL distilled water and mixed properly by using a Homogenizer (SHG-15D, SciLab, Seoul, Korea) for 3 min. Then, the homogenate was collected for measuring pH using an Ohaus Starter 2100 pH meter (Seoul, Korea).

The VBN value of smoked HDPS was estimated by using a Conway micro diffusion method [17].

According to the previous study of Oğuzhan Yildiz [26], a 5 g smoked HDPS sample was homogenized with a 12.5 mL TCA solution (20% trichloroacetic acid in 2 M phosphoric acid). The absorbance was measured at 530 nm wavelength using a microplate reader (SPECTROstar Nano, Newtown, UK). The lipid peroxidation values were expressed as mg of malonaldehyde per kg of processed HDPS fillet.

### 2.6. Analysis of TMAO Level

The TMAO content in both smoked and raw HDPS was quantitatively determined using a Gas Chromatography-Mass Spectrometry (GC/MS) analysis. The analysis method of TMAO content was followed by the previous study of Mohibbullah et al. [16]. The TMAO content of raw and smoked HDPS was identified and quantified by comparing it with the standard compound (Sigma-Aldrich, St. Louise, MO, USA).

### 2.7. Analysis of Nutritional Composition

To quantify the nutritional quality of hot smoked HDPS fillet, the standard method of AOAC [27] was followed and performed at the Traditional Microorganism Resources Center, Keimyung University, Daegu, Korea.

### 2.8. Analysis of Fatty Acids

Gas chromatography (Shimadzu Corp., Kyoto, Japan) with a flame ionization detector (GC-FID) was used for the quantification of fatty acid methyl esters (FAMEs). Supelco SP-2560 column (100 m × 0.25 mm × 0.25 μm) with an oven temperature of 240 °C, which increased from 100 °C to 200 °C at a flow rate of 3.5 °C/min, was used for the separation of FAMEs from fish fillets. The helium gas was used as a carrier at a split ratio of 1:50, and the obtained FAME signals were analyzed and validated by comparing the retention time of standard fatty acids [12].

### 2.9. Analysis of Benzo[a]pyrene (BaP)

The Bap content was analyzed using a High-Performance Liquid Chromatography (HPLC; LC-20A, Shimadzu Corp.), with a fluorescence detector (RF-20A, Shimadzu Corp.) of excitation and an emission wavelength of 294 nm and 404 nm, respectively, according to the instruction of the Ministry of Food and Drug Safety (MFDS) [28] and performed at Hankyul Institute of Technology Analysis (Sacheon-si, Gyeongsangnam-do, South Korea).

### 2.10. Statistical Analysis

All of the experimental results were expressed as the mean ± standard deviation (std). The statistical analysis was performed using an IBM SPSS software version 20.0 (IBM, Corp., New York, USA), followed by one-way analysis of variance (ANOVA) with LSD and Duncan’s multiple range tests. The statistical significance in the analysis was deemed at *p* < 0.05.

## 3. Results

### 3.1. Effect of Hot Smoking with Different Sawdust Materials on Odor Intensity and Sensory Characterization

**Figure 1:**
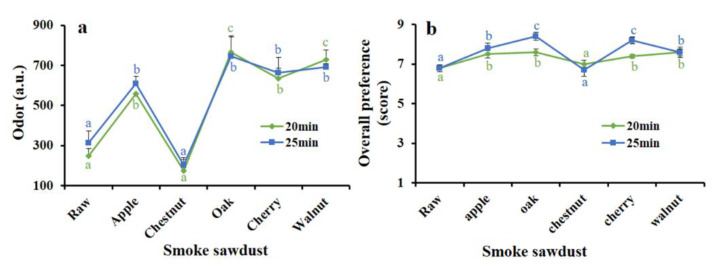
Use of different sawdust materials on the improvement of sensory attributes of (**a**) odor (a.u.) and (**b**) overall preference (hedonic score) at 20 and 25 min of hot smoking times of half-dried Pacific Saury (HDPS). Data represent the mean ± std of 10 observations, where groups not sharing a letter are expressed as significantly different (*p* < 0.05).

The sawdust materials help to develop the color and flavor of the HDPS smoked product. Instrumental odor intensity was observed and found to be significantly higher (*p* < 0.001) while using Oak sawdust-smoke followed by cherry, walnut, and apple sawdust-smoke at two different smoking times of 20 and 25 min (Figure 1a). The results of overall preferences while using different sawdust materials were significantly (*p* < 0.001) increased, except for chestnut, as compared with raw HDPS (Figure 1b). The results suggested that Oak sawdust was found to be the most appropriate smoking material for developing sensory quality in HDPS.

### 3.2. Optimization of Smoking Treatment

**Figure 2:**
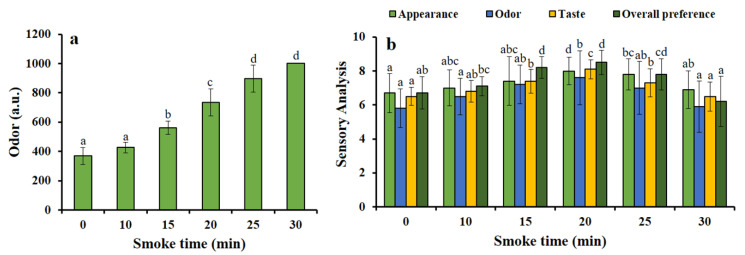
Different smoking times on the changes of (**a**) odor (a.u.) and (**b**) sensory quality attributes of hot smoked HDPS. Data represent the mean ± std of 10 observations, where groups not sharing a letter are expressed as significantly different (*p* < 0.05).

The instrumental odor intensity was found to be significantly (*p* < 0.001) higher with the increase in smoking time (Figure 2a). The optimum smoke time was perceived by evaluating the hedonic scale (0–9) based on sensory analysis of smoked HDPS. We found that smoking times of 20 and 25 min increased sensory attributes (appearance, odor, taste, and overall preference) significantly (*p* < 0.01 and/or *p* < 0.001) (Figure 2b). For further validation, we considered whether those smoking times could maintain proper physicochemical properties in HDPS, and, therefore, we kept continuing hot smoking treatment time-dependently for further experiments.

### 3.3. Hot Smoking Treatment Improved Physical Properties of HDPS

#### 3.3.1. Changes of Weight Loss by the Smoking Time

**Figure 3:**
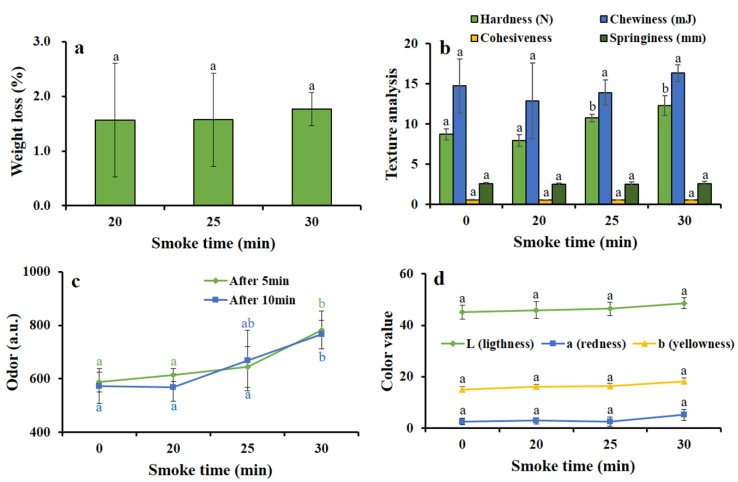
Different smoking times on the effects of (**a**) weight loss, (**b**) texture analysis, (**c**) odor (a.u.), and (**d**) color value of hot smoked HDPS. Data represent the mean ± std of 10 observations, where groups not sharing a letter are expressed as significantly different (*p* < 0.05).

The weight loss of a fish product indicates the removal of moisture from the fish, which is ultimately responsible for reducing the firmness of fish products. A non-significant difference was found in the weight loss analysis of hot smoked HDPS groups of different smoking times (Figure 3a).

#### 3.3.2. Changes of Instrumental Texture Analysis by Smoking Time

Textural properties of hot smoked HDPS product were analyzed instrumentally and the increase in hardness between 25 and 30 min smoking time was found to be significant (*p* < 0.05 and *p* < 0.001), and other textural parameters such as Chewiness, Springiness, and Cohesiveness remained the same and non-significant along with the increased smoking time (Figure 3b).

#### 3.3.3. Changes of Odor Intensity by the Smoking Time

Odor intensity is considered as a critical parameter for increasing the acceptability of fishery products to the consumer. In this study, odor intensity was calculated at 5 and 10 min intervals at different smoking periods of 0, 20, 25, 30 min. The study found odor intensity increased with increasing smoke time and was significantly higher (*p* < 0.01) at 30 min of smoking time (Figure 3c).

#### 3.3.4. Changes of Color Value by the Smoking Time

The color of fish products is one of the important attributes to positively attract consumer preference. The color value (lightness, redness, and yellowness) of HDPS fillet remained constant, indicating that the hot smoke had no negative influence on HDPS fillet (Figure 3d).

### 3.4. Hot Smoking Treatment Improved Biochemical Features of HDPS

#### 3.4.1. Changes of TBC by the Smoking Time

**Figure 4:**
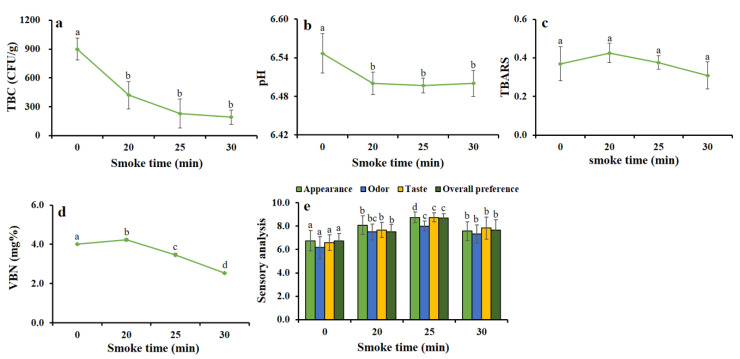
Different smoking times on the improvement of (**a**) TBC (CFU/g), (**b**) pH, (**c**) TBARS, (**d**) VBN (mg%), and (**e**) sensory evaluation of hot smoked HDPS. Data represent the mean ± std of 10 observations, where groups not sharing a letter are expressed as significantly different (*p* < 0.05).

The bacterial biomass is responsible for the spoilage of fish products. Smoking treatment with wood-smoke has antibacterial agents that could effectively suppress bacterial growth. The study found the total bacterial count was significantly (*p* < 0.01 and *p* < 0.001) decreased while increasing the smoke time of 20, 25, and 30 min (Figure 4a).

#### 3.4.2. Changes of pH Value by the Smoking Time

The fish product quality depends on the pH value. We found that the pH value significantly (*p* < 0.05) decreased with increased smoke time (Figure 4b).

#### 3.4.3. Changes of TBARS Value by the Smoking Time

The TBARS value indicates the degradation of fat in smoked HDPS products. The value of TBARS non-significantly increased up to the smoke time of 20 min, and afterward, it gradually decreased because of increasing the smoke time (Figure 4c).

#### 3.4.4. Changes of VBN Value by the Smoking Time

The VBN value is used as an indicator for the evaluation of the degree of spoilage in fish and fishery products. In Figure 4d, initially, the VBN value at 20 min smoking time increased non-significantly, while that of 25 and 30 min of smoking treatment decreased the VBN value significantly (*p* < 0.001).

#### 3.4.5. Changes of Sensorial Characteristics by the Smoking Time

The sensory evaluation was performed by well-trained panelists. The sensory characteristics of appearance, odor, taste, and overall preference were significantly (*p* < 0.001) increased at the smoke times of 20, 25, and 30 min (Figure 4e). Among the smoke time, better sensory scores were observed at 25 min of smoking time. Therefore, we optimized and chose, based on the time-dependent results, this 25-min smoking time for further experiments.

### 3.5. Hot Smoking Preserved Biochemical Characteristics of HDPS during Storage Condition

#### 3.5.1. Changes of Microbiological Activity

Temperature is an important determinant for bacterial growth promotion in fishery products during storage period. The hot smoked HDPS fillet was kept at two different temperatures (10 °C and 15 °C) for 0 to 32 days. Bacterial growth was not observed at a storage temperature of 10 °C, but at 15 °C temperature, bacterial growth appeared after 18 days of storage period (Table 1). Moreover, the coliform count was found absent in both storage temperatures. The results of microbial growth indicate that hot smoking with Oak sawdust might have the potential to suppress bacterial growth most effectively at a storage temperature of 10 °C, and, thereby, it amazingly extended the shelf life of the HDPS product up to 32 days.

#### 3.5.2. Changes of VBN Level

**Figure 5:**
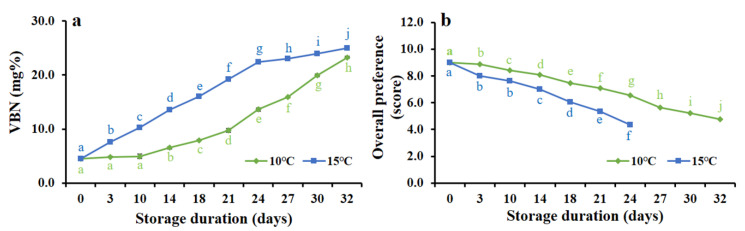
Consequences of storage time on (**a**) volatile basic nitrogen (VBN) and (**b**) sensory evaluation (overall preference) of hot smoked HDPS at two different storage temperature of 10 °C and 15 °C Data represent the mean ± std of 10 observations, where groups not sharing a letter is expressed as significantly different (*p* < 0.05).

The VBN value of smoked HDPS was determined intermittently in two storage temperatures at 10 °C and 15 °C from 0 to 32 days. The VBN value was found to have a significantly (*p* < 0.001) increasing trend and values ranging between 4.5 and 23.3 (mg%), and 4.5 and 25.0 (mg%) at 10 °C and 15 °C storage temperatures, respectively (Figure 5a).

#### 3.5.3. Changes of Overall Preferences

The sensory evaluation (overall preference) of hot smoked HDPS was found to have significantly (*p* < 0.001) declined with the extension of storage time at two different storage conditions of 10 °C and 15 °C (Figure 5b). The results indicate that the overall preference remained acceptable even after 30 days of storage time at 10 °C, and on the other hand, at 15 °C storage product remained safe up to 21 days and then decreased its sensorial attributes. The study reveals that hot smoked HDPS at 10 °C storage temperature provided better product quality in extending the storage duration.

### 3.6. Hot Smoking Restricted TMAO Level

**Figure 6:**
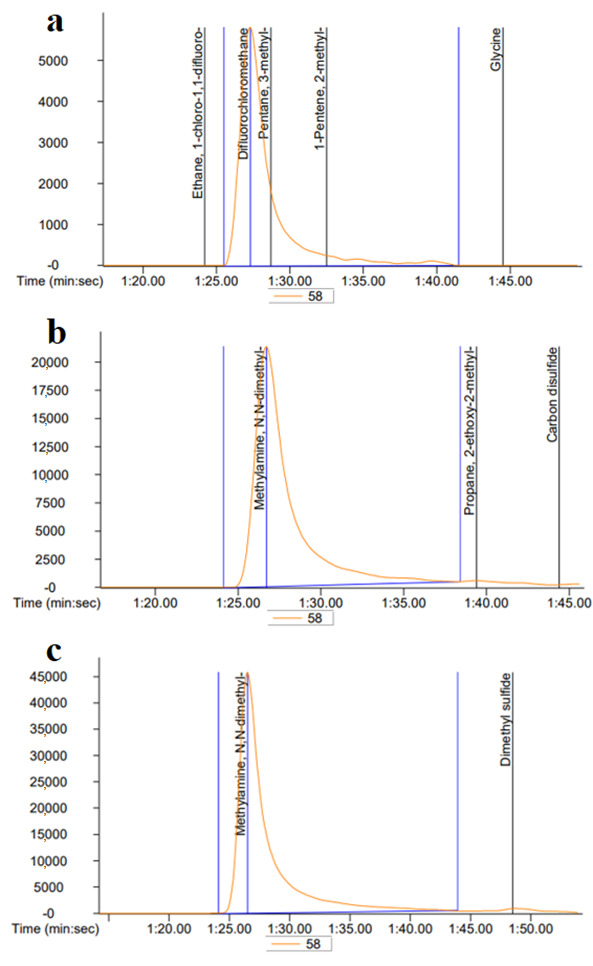
The trimethyl N-oxide (TMAO) was identified and quantified by GC/MS analysis. The representative chromatograms of (**a**) standard TMAO, (**b**) raw HDPS, and (**c**) hot smoked HDPS. The TMAO was identified and quantified by the reference TMAO injecting into the GC/MS. The TMAO spikes in the chromatograms appeared at 1 min 25 s of retention time among different treatment groups.

The TMAO concentration is one of the predominant factors for decreasing the freshness of fishery products, when it introduces, which undergoes an off-odor of the product, and therefore, it is considered as a key determinant for the degree of spoilage. Here, we determined whether the TMAO level had been suppressed using a selective Oak sawdust-mediated hot smoking of HDPS. The TMAO was identified and quantified by injecting the reference TMAO reagent into the GC/MS. The resultant peak area appeared at the same retention time of 1 min 25 s among different treatment groups (Figure 6). Following the results of TMAO content by GC/MS analysis, hot smoked HDPS contained a low level of TMAO which was 1.13 ± 0.17 μg/100 g, while raw HDPS was 2.16 ± 0.28 μg/100 g (triplicate measurement), indicating that hot smoking with Oak sawdust had a suppressive effect on TMAO production in HDPS.

### 3.7. Hot Smoking Preserved Nutritional Quality

The nutritional value of smoked fish and fishery products may vary upon changing of handling, processing, and storage conditions. In analyzing the nutritional quality of hot smoked HDPS fillet, the total calories were 479.885 kcal/100 g, in which the major components such as carbohydrates, lipids, (crude, trans and saturated fat) and proteins (crude) were 0.316, 46. 897, and 34.720 g/100 g, respectively, (Table 2). Hot smoked HDPS possessed higher mineral contents sodium (208.829 mg/100 g), potassium (369.862 mg/100 g), calcium (60.013 mg/100 g), and iron (2.05 mg/100 g).

### 3.8. Hot Smoking Preserved Beneficial Fatty Acids

We identified and quantitated the fatty acids from hot smoked HDPS products and precisely elucidated on its positive correlation between hot smoking process, and its valuable and functional fatty acid contents. We, therefore, found 36 fatty acids in hot smoked HDPS and compiled them into three groups as monounsaturated fatty acids (MUFAs), polyunsaturated fatty acids (PUFAs), and saturated fatty acids (SFA). Among them, the contents of MUFAs, PUFAs, and SFA were 6.37 g/100 g, 5.89 g/100 g, and 4.36 g/100 g, respectively (Table 3). Of 36 fatty acids in HDPS identified and quantified, the lead fatty acid was found to be docosahexaenoic acid (DHA; 3.03 g/100 g) followed by erucic acid (2.79 g/100 g), cis-11-eicosenoic acid (1.81 g/100 g), eicosapentaenoic acid (EPA; 1.17 g/100 g), and oleic acid (OLA; 1.04 g/100 g). In addition, HDPS with hot smoking contained a higher level of omega-3 (DHA and EPA) fatty acids, which are attributable to the beneficial health effects on humans.

### 3.9. Hot Smoking Restricted BaP Content

In polycyclic aromatic hydrocarbons (PAHs), the BaP is one of the most promising organic compounds involved in the mutagenic or carcinogenic processes, and is released from wood materials whenever hot smoking is performed. We determined whether Oak sawdust-mediated hot smoking of HDPS possessed a BaP compound. The result obtained from HPLC analysis was marked as no detection of BaP compounds while injecting the samples with reference BaP at standard dilution conditions. Since the limit of quantification of BaP was 0.9 μg/kg, the following injecting condition also did not allow for the quantification of the sample. It suggested that the content of BaP in hot smoked HDPS was shown to be acceptable and safe for human consumption.

## 4. Discussions

Fish and fishery products have been associated with a healthy balanced diet because of their variety of valuable nutrients, including protein, lipid, and especially, long-chain omega-3 and -6 polyunsaturated fatty acids (n-3 and -6 PUFAs), as well as vitamins (e.g., A, D and B) and other minerals (e.g., iodine, selenium) [29]. Fish is a highly perishable food items because it contains a high concentration of unsaturated fatty acids, which initially are attacked by the oxygen, as a result, it gives rise to rancidity with off-odor, especially observed in smoked and dried fish, decreasing the shelf life by allowing collectively implicated enzymatic and bacterial actions to trigger various chemical changes in texture, appearance, taste, and overall qualities [30]. The present study was aimed at investigating development of a hot smoking method as an effective processing and preservation tool to improve the sensorial characteristics, physicochemical properties, and subsequent extension of the shelf life of HDPS, having a quality of improving health functions to the end-point consumers.

One of the easiest and simplest methods for evaluating the consumer products is sensory evaluation, which is associated with various sensorial parameters such as color, odor, taste, and overall preference; the scores of each attribute are based on a 9-point hedonic scale [22]. In the present study, when different sawdust materials (apple, oak, cherry, chestnut, and walnut) were used in hot smoking (70 °C) process of HDPS fillet, time-dependently ranging from 0 to 30 min of smoking time, the study found highly acceptable sensory characteristics at a 25-min smoke time. In compliance with this result, significant sensory impacts were observed when hot smoking was performed with Wels catfish [31]. It is evident that sawdust material released several volatile compounds while smoking, among which phenols are likely to be the compounds predominantly responsible for developing color and aroma of food items [32]. The results obtained, the Oak sawdust material possessed the best sensory quality in HDPS, which is similar to the study of Mohibbullah et al. [16].

The firmness of fish products is one of the most significant quality parameters pertinent to consumer preference [33]. The processed HDPS remained unchanged in weight loss determination at a different smoke time (20, 25, and 30), indicating good quality smoked products for consumers. The texture profile of a seafood product is one of the most significant physical quality attributes, to which, a consumer can easily perceive to evaluate the hardness, springiness, adhesiveness, and gumminess of a given food [12]. In HDPS, all textural characters remained stable and considerable at different smoking times. The study showed that texture profile persisted steadily at the different smoke times (0, 20, 25, and 30 min), which was more or less similar to the previous study by Mohibbullah et al. [16], who found springiness, cohesiveness, and hardness remained stable, but only chewiness increased far less after 15 min of smoking time. The odor intensity and color value play a significant role in the acceptance or rejection of fish products by the consumer [34]. The instrumental odor and color value remained unchanged for a smoke time of 25 min, and the results corresponded to the previous report of Zzaman et al. [35].

It is evident that hot smoking treatment inhibits the growth of microorganisms [11], and in line with the present study, which suggested that a significant (*p* < 0.001) decrease in bacterial growth was found when the hot smoking time was 25 min, it is likely that volatile materials existed in the wood smoke [14,36]. Positive and favorable consequences were observed in hot smoked HDPS; desirable and considerable levels of pH, TBARS, and VBN were found at 25 min of smoking time, in comparison with other studies, which found the similar effects [3,16,17,26]. In storage conditions at 10 °C and 15 °C, the VBN value was found an increasing trend, but within an acceptable limit, which was similar to the reports of previous studies [16,26,37]. In addition, the overall sensorial impact of HDPS was significantly decreased with increasing storage time, however, 10 °C storage conditions showed a better acceptance by panelists even after 30 days but 15 °C did not. The bacterial growth on hot smoked HDPS (TBC and coliform) was not seen at 10 °C after a period of 32 days of storage. It was reported that a combined treatment of superheated steam and smoking on squid showed preventive effects against microbial growth while storing at 10 °C [12].

Conversion of TMAO into TMA (Trimethylamine) in fish after death usually happens due to the existing enzymes and bacterial action [17], and its quantification is an approach of evaluating fish and fishery product quality with a maximum allowable limit as <10–15 mg/100 g [34,38]. By applying hot smoke with Oak sawdust in HDPS, the TMAO content was significantly reduced when compared with raw HDPS, a similar effect as seen by the previous report [16,17]. Moreover, the nutritional composition of hot smoked HDPS remained high after treatment, indicating that there was less possibility of nutritional degradation during the treatment when compared with previous literature [38]. One of the salient features of using hot smoking with Oak sawdust was that HDPS possessed higher concentrations of fatty acids including MUFAs and PUFAs. Fatty acid is an important nutritional component that provides promising health benefits [39]. The study revealed a higher amount of omega-3 fatty acids, particularly DHA and EPA, found in hot smoked HDPS products, which are known to be effective in mitigating the onset of neurodegenerative complications in central nervous system neurons, cardiovascular disease progression, and arthritis [40,41,42]. PAHs are generally formed from incomplete combustion of organic matter such as wood materials while smoking and considered to be potential health hazards, in which BaP represents an effective genotoxic and carcinogenic to humans [43]. In considering food safety and public health, there need for further evaluation of the content of BaP in hot smoked HDPS. We determined the content of BaP in hot smoked HDPS and the result suggested that there was no detectable limit for BaP content (<0.9 μg/kg), indicating it to be acceptable and safe for human consumption [43,44]. A similar study found that BaP was not detected in commercial smoked sardine, silver carp, squid, or tuna [45].

## 5. Conclusions

Hot smoking (70 °C) with selective Oak sawdust improved the sensorial and physicochemical attributes of HDPS. The inhibitory effects of HDPS on bacterial growth and TMAO content were observed in hot smoked HDPS fillet. Moreover, higher nutritional composition was obtained, of which beneficial polyunsaturated fatty acids, including DHA, EPA, and OLA, were remarkably high in our study. With all these preferential attributes in HDPS, it could become a product of premium quality to the consumer, with a shelf life that could be extended up to 32 days at 10 °C storage conditions without concerning any quality loss. The present hot smoking process had no effects on PAHs accumulation, particularly BaP in HDPS fillets, indicating a safer food item for consumers. It is believed that this processing technique has great feasibility to preserve food products with both nutritional and functional characteristics in the future, particularly, for the pacific saury *Cololabis saira.*

## Figures and Tables

**Table 1:** Microbial activity of hot smoked HDPS during storage period.

Storage Duration (Days)	Bacteria (CFU/g)		Coliform	
	10 °C	15 °C	10 °C	15 °C
0	0	0	0	0
3	0	0	0	0
10	0	0	0	0
14	0	0	0	0
18	0	21.67	0	0
21	0	36.67	0	0
24	0	45.00	0	0
27	0	-	0	-
30	0	-	0	-
32	0	-	0	-

**Table 2:** Nutritional composition of hot smoked HDPS.

Test Items	Unit	Test Results
Calories	kcal/100 g	479.885
Sodium	mg/100 g	208.829
Carbohydrate	g/100 g	0.316
Sugars	g/100 g	0.181
Crude fat	g/100 g	37.749
Trans fat	g/100 g	0.131
Saturated fat	g/100 g	9.017
Cholesterol	mg/100 g	51.588
Crude protein	g/100 g	34.720
Potassium	mg/100 g	369.862
Calcium	mg/100 g	60.013
Iron	mg/100 g	2.050
Vitamin D	mg/100 g	ND

**Table 3:** Fatty acid profile of hot smoked HDPS.

Fatty Acids		Hot Smoked HDPS (g/100 g)
Butyric acid	C4: 0	0.00
Caproic acid	C6: 0	0.00
Caprylic acid	C8: 0	0.00
Capric acid	C10: 0	0.00
Lauric acid	C12: 0	0.01
Tridecanoic acid	C13: 0	0.01
Myristic acid	C14: 0	1.17
Pentadecanoic acid	C15: 0	0.10
Palmitic acid	C16: 0	2.35
Stearic acid	C18: 0	0.43
Margaric acid	C17: 0	0.20
Heneicosanoic acid	C21: 0	0.00
Behenic acid	C22: 0	0.01
Arachidic acid	C20: 0	0.08
Tricosanoic acid	C23: 0	0.00
Lignoceric acid	C24: 0	0.00
**∑ SFA**		**4.36**
Myristoleic acid	C14: 1	0.01
Pentadecenoic acid	C15: 1	0.00
Palmitoleic acid	C16: 1	0.55
Margaroleic acid	C17: 1	0.00
Elaidic acid	C18: 1 n-9t	0.03
Oleic acid	C18: 1 n-9c	1.04
Cis-11-Eicosenoic acid	C20: 1 n-9	1.81
Erucic acid	C22: 1 n-9	2.76
Nervonic acid	C24: 1 n-9	0.17
**∑ MUFA**		**6.37**
Linolelaidic acid	C18: 2 n-6t	0.19
Linoleic acid	C18: 2 n-6c	0.32
ϒ-Linolenic acid	C18: 3 n-6	0.02
Cis-11, 14-Eicosenoic acid	C20: 2 n-6	0.62
Docosadienoic acid	C22: 2 n-6	0.13
Cis-11, 14, 17-Eicosenoic acid	C20: 3 n-3	0.07
α-Linolenic acid	C18: 3 n-3	0.23
Dihomo ϒ-Linolenic acid	C20: 3 n-6	0.03
Arachidonic acid	C20: 4 n-6	0.08
Eicosapentaenoic acid	C20: 5 n-3	1.17
Docosahexaenoic acid	C22: 6 n-3	3.03
**∑ PUFA**		**5.89**

SFA: Saturated Fatty Acid; MUFA: Mono Unsaturated Fatty Acid; PUFA: Poly Unsaturated Fatty Acid.
